# Protective Effects of Recombinant Human Soluble Thrombomodulin on Lipopolysaccharide-Induced Acute Kidney Injury

**DOI:** 10.3390/ijms21072519

**Published:** 2020-04-05

**Authors:** Yuji Nozaki, Jinhai Ri, Kenji Sakai, Kaoru Niki, Masanori Funauchi, Itaru Matsumura

**Affiliations:** Department of Hematology and Rheumatology, Kindai University Faculty of Medicine, Osaka-sayama, Osaka 589-8511, Japan; jinhai@med.kindai.ac.jp (J.R.); kenji-s-101@med.kindai.ac.jp (K.S.); komyodai9300@yahoo.co.jp (K.N.); mn-funa@med.kindai.ac.jp (M.F.); kougen@med.kindai.ac.jp (I.M.)

**Keywords:** acute kidney injury, sepsis, apoptosis, cytokine

## Abstract

Thrombomodulin (TM) is a single transmembrane, multidomain glycoprotein receptor for thrombin, and is best known for its role as a cofactor in a clinically important natural anticoagulant pathway. In addition to its anticoagulant function, TM has well-defined anti-inflammatory properties. Soluble TM levels increase significantly in the plasma of septic patients; however, the possible involvement of recombinant human soluble TM (rTM) transduction in the pathogenesis of lipopolysaccharide (LPS)-induced nephrotoxicity, including acute kidney injury (AKI), has remained unclear. Mice were injected intraperitoneally with 15 mg/kg LPS. rTM (3 mg/kg) or saline was administered to the animals before the 3 and 24 h LPS-injection. At 24 and 48 h, blood urea nitrogen, the inflammatory cytokines in sera and kidney, and histological findings were assessed. Cell activation and apoptosis signal was assessed by Western blot analysis. In this study using a mouse model of LPS-induced AKI, we found that rTM attenuated renal damage by reducing both cytokine and cell activation and apoptosis signals with the accumulation of CD4+ T-cells, CD11c+ cells, and F4/80+ cells via phospho c-Jun activations and Bax expression. These findings suggest that the mechanism underlying these effects of TM may be mediated by a reduction in inflammatory cytokine production in response to LPS. These molecules might thereby provide a new therapeutic strategy in the context of AKI with sepsis.

## 1. Introduction

The incidence of acute kidney injury (AKI) ranges from 5% in all hospitalized patients to 30–50% in intensive care units [1]. AKI is a frequent complication of sepsis, and contributes substantially to the high morbidity and mortality seen with sepsis [2,3]. This lethality has been thought to result from severe inflammation during infection. Anti-inflammatory agents such as interleukin (IL)-1 receptor antagonists and neutralizing antibodies against tumor necrosis factor (TNF) have been shown to be efficacious for the treatment of an animal model of sepsis [4,5]. Infiltrating cells have also been indicated as playing important roles in the initiation and progress of tubule dysfunction and structural injury in AKI [6] by inhibiting infiltration and inflammatory cytokines by lymphocytes [7] or macrophages [8], in turn decreasing tubule damage.

Thrombomodulin (TM) is a single transmembrane, multidomain glycoprotein receptor for thrombin that is best known for its role as a cofactor in a clinically important natural anticoagulant pathway [9], and which provides a definitive molecular bridge that links inflammation and coagulation. In addition to its anticoagulant function, TM has well-defined anti-inflammatory properties [10,11]. TM is expressed predominantly by vascular endothelial cells, but also by several other cell types, including neutrophils, cells of the synovial lining, as well as monocytes and osteoblasts [12,13,14,15]. TM activates protein C and produces anti-inflammatory effects, such as suppression of TNF by the inhibition of macrophage activation [16]. Recombinant human soluble TM (rTM) has been approved for the treatment of disseminated intravascular coagulation in Japan [17]. rTM is composed of an *N*-terminal lectin-like domain and a domain rich in *O*-glycosylation. The *N*-terminal lectin-like domain is an important inactive pro-inflammatory high-mobility group box1 protein [18], whereas the fourth, fifth, and sixth epidermal growth factor-like domains of soluble rTM are critical to form a high-affinity complex with thrombin and to activate anticoagulant protein C (APC) [18], which is known to inhibit the activation of monocytes and macrophages [16,19]. However, the role of TM in lipopolysaccharide (LPS)-induced AKI remains unclear. We hypothesized that when injured kidneys became hypersensitive to LPS, a component of Gram-negative bacteria, the levels of circulating inflammatory cytokines such as IL-18, TNF, and interferon-gamma (IFN-γ) could be elevated. Such an increase may then result in a worsening of the AKI, resulting in further damage to renal function. In the present study, we evaluated the effect of rTM on survival rate and systemic immune response in a mouse model of LPS-induced AKI.

## 2. Results

### 2.1. Experimental Schedule

**Figure 1:**
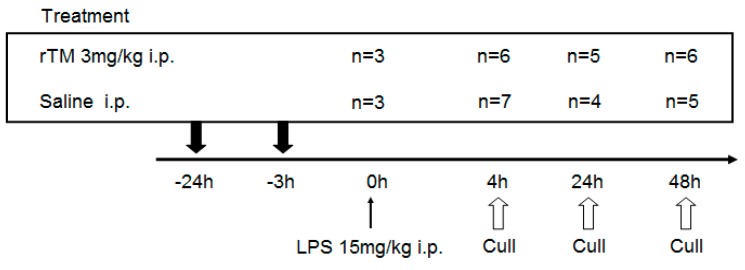
Experimental protocol of the lipopolysaccharide (LPS) model. A total of 53 C57BL/6 mice were injected intraperitoneally with 15 mg/kg of lipopolysaccharide (LPS) at 0 h for the evaluation of survival rate and for measurements. Groups of mice were given either recombinant human soluble thrombomodulin (rTM) (3 mg/kg) as the rTM treatment or saline as control, twice (−24 and −2 h), by intraperitoneal injection. Mice were culled at the following times (control groups and rTM treatment, respectively): 4 (*n* = 7 and 6), 24 (*n* = 4 and 5), and 48 h (*n* = 5 and 6). Specimens were collected on an hourly schedule.

The experimental schedule is shown in Figure 1. A total of 53 C57BL/6 mice were injected intraperitoneally (i.p.) with 15 mg/kg of LPS at 0 h for the evaluation of survival rate and for measurements. Groups of mice were given either rTM (3 mg/kg) as the rTM treatment or saline as control, twice (−24 and −2 h), by intraperitoneal injection. Mice were culled at the following times: 4, 24, and 48 h. Specimens were collected on an hourly schedule.

### 2.2. Recombinant Human Soluble TM after LPS-Induced AKI Model

**Figure 2:**
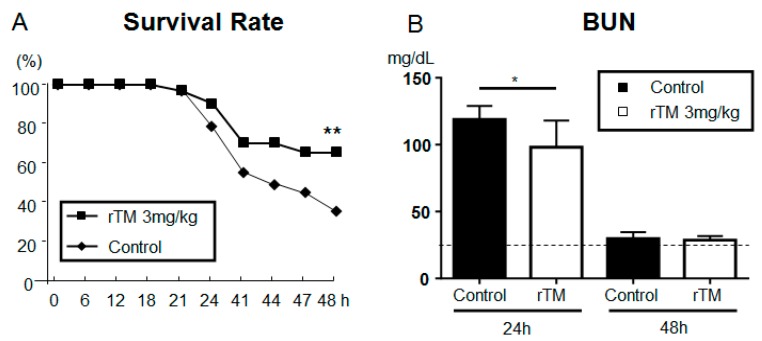
Effect of recombinant human soluble thrombomodulin (rTM) on the survival and blood urea nitrogen (BUN) level in the development of acute kidney injury after lipopolysaccharide (LPS) injection. (**A**) Survival of control and rTM-treated mice subjected to sepsis by LPS-injection. Mice were evaluated until 48 h after LPS-injection (control, *n* = 20; rTM, *n* = 20; ** *p* < 0.01). (**B**) Renal function of control and rTM-treated mice at 0 (control, *n* = 5; rTM, *n* = 5), 24 (control, *n* = 18; rTM, *n* = 16), and 48 h (control, *n* = 8; rTM, *n* = 12; ** *p* < 0.01) before and after LPS-injection, assessed by BUN levels. The data are the mean values ± standard error of the mean (* *p* < 0.05, control versus rTM-treated mice). Dotted lines represent mean values from saline-treated mice without LPS.

We observed that the rTM-treatment mice had a significantly higher survival rate at 48 h (control 35.0% versus rTM 65.0%) (Figure 2A). The LPS-induced rise in blood urea nitrogen (BUN) levels at 24 h was also significantly decreased in the rTM-treatment mice (control 118.9 ± 3.6 versus rTM 98.0 ± 6.7 mg/dL) (Figure 2B). At 48 h, the BUN levels in all mice had decreased to the same level as that observed prior to LPS-injection (control 29.9 ± 2.4 versus rTM 28.7 ± 1.7 mg/dL).

### 2.3. Serum Biomarkers in AKI

**Figure 3:**
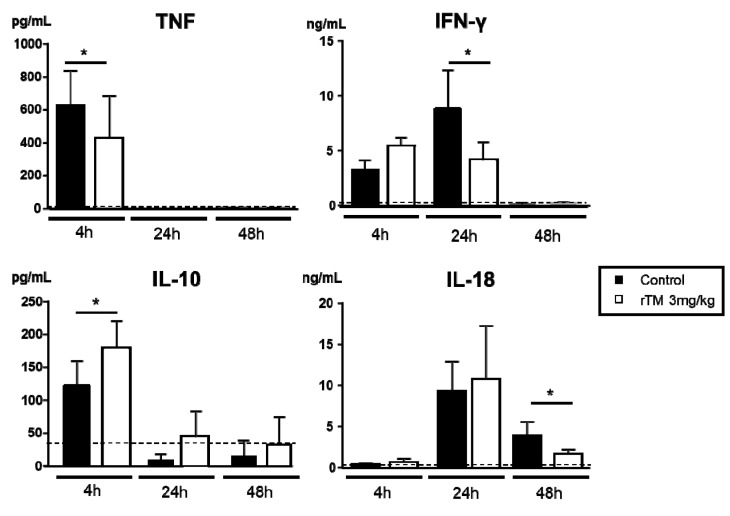
Effect of recombinant human soluble thrombomodulin (rTM) on the production of inflammatory cytokines after lipopolysaccharide (LPS) injection. Serum tumor necrosis factor (TNF), interferon (IFN)-γ, interleukin (IL)-10, and IL-18 production was measured as a biomarker of AKI at 0 (control and rTM, *n* = 3), 4 (*n* = 7 and 6), 24 (*n* = 4 and 5), and 48 h (*n* = 5 and 6) before and after LPS-injection. The data are the mean values ±standard error of the mean (* *p* < 0.05, control versus rTM-treated mice).

The serum levels of TNF, IFN-γ, IL-10, and IL-18 were measured as biomarkers in AKI (Figure 3). The cytokine levels of IL-10 and TNF, and IFN-γ and IL-18, peaked at 4 and 24 h. IL-18 had greatly decreased at 48 h after LPS-injection. The serum TNF levels at 4 h, IFN-γ levels at 24 h, and IL-18 levels at 48 h were significantly decreased in rTM-treatment mice compared to the corresponding levels in control mice (control versus rTM for all of the following): TNF, 628.2 ± 37.1 versus 428.0 ± 49.1 pg/mL; IFN-γ, 8.9 ± 2.4 versus 4.1 ± 8.4 ng/mL; IL-18, 3.9 ± 8.4 versus 1.7 ± 2.5 ng/mL). Conversely, IL-10 levels at 4 h were significantly increased in rTM-treatment mice compared to the corresponding levels in control mice (control 122.8 ± 11.3 versus rTM 180.0 ± 23.7 pg/mL).

### 2.4. Infiltration of CD4+ T-Cells, CD11c+ Cells, Macrophages, and Neutrophils in the Kidney

**Figure 4:**
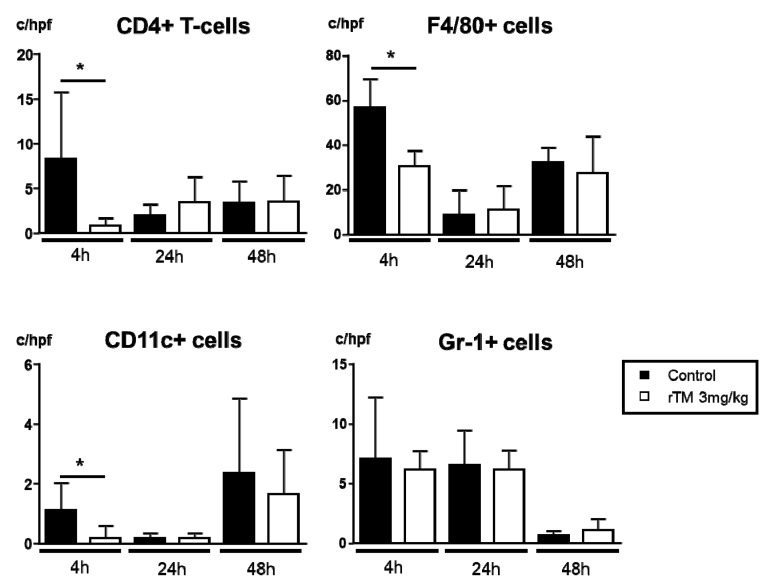
Effect of recombinant human soluble thrombomodulin (rTM) on the accumulation of inflammation cells in the interstitium after lipopolysaccharide (LPS) injection. The accumulation of CD4+ T-cells, and F4/80^+^, CD11c+, and Gr-1+ cells in the interstitium at 0 (control and rTM, *n* = 3), 4 (*n* = 7 and 6), 24 (*n* = 4 and 5) and 48 h (*n* = 5 and 6) before and after LPS-injection. The data are the mean ±standard error of the mean (* *p* < 0.05, cell numbers in control versus rTM-treated mice).

We investigated the infiltration of inflammation cells, that is, CD4+ T-cells, macrophages, CD11c+ cells, and neutrophils in the renal interstitium (Figure 4). The number of interstitial CD4+ T-cells (control 8.3 ± 3.3 versus rTM 0.8 ± 0.3 cells/hpf), macrophages (F4/80) (control 56.7 ± 6.3 versus rTM 30.6 ± 4.0 cells/hpf), and CD11c+ cells (control 1.1 ± 0.3 versus rTM 0.2 ± 0.2 cells/hpf) in control mice were significantly increased compared those in the rTM-treatment mice at 4 h. On the other hand, the number of interstitial neutrophils was not significantly different between the two groups during the experimental period.

### 2.5. Renal mRNA Expression in LPS-Induced AKI

Inflammatory cytokines and chemokines are well described in LPS-induced AKI [20]. We therefore measured mRNA expression in the kidney by conducting real-time reverse transcription-polymerase chain reaction (RT-PCR) analysis (Table 1). The cytokines and chemokine mRNA expression (TNF, chemokine (C-C motif) ligand 2/monocyte chemoattractant protein-1 (CCL2/MCP-1), and IFN-γ) were reduced in the rTM-treatment group at 4, 24, and 48 h, respectively. At 48 h, these cytokines and chemokines were downregulated and revealed no significant differences between the control and rTM-treatment mice. Conversely, IL-10 mRNA expression increased in the rTM-treatment mice at 48 h. Here, we evaluated the mRNA expression for two Th-cell subset transcription factors, T-bet (Th1) and GATA3 (Th2), in kidney mRNA from each group of mice. The T-bet expression at 4 h after LPS-injection was reduced in rTM-treatment mice compared to that of the control mice, but no changes in GATA3 expression were observed in either group. Therefore, rTM administration shifted systemic responses away from the Th1 phenotype. There were no significant differences in Toll-like receptor4 (TLR4) mRNA expression between the control and rTM-treatment mice.

### 2.6. Renal Kidney Injury Molecule-1 Expression

**Figure 5:**
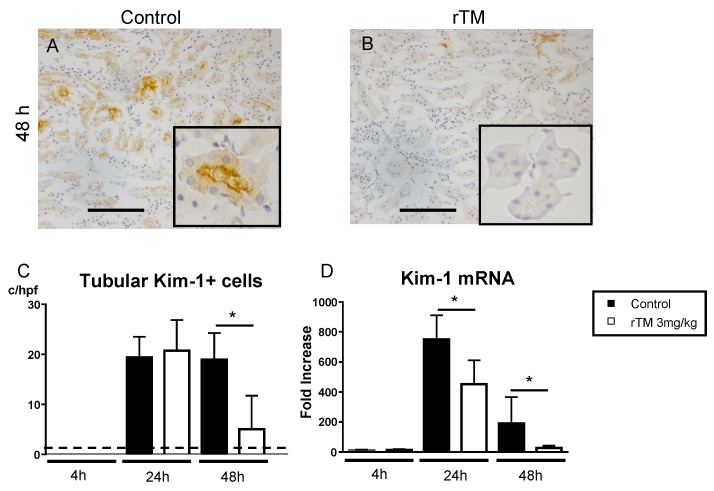
Expression of Kim-1 (kidney injury molecule-1) in the kidney. Representative photograph of Kim-1 expression in the kidney after lipopolysaccharide (LPS) injection (**A**,**B**). In saline-treated control mice, Kim-1-positive tubules were present in the kidney (**A**). Kim-1-positive tubules were significantly visible in the rTM-treated mice, whereas they were not visible in the control mice (**B**). The number of Kim-1-positive tubules in 10 × 400 fields (**C**). Expression of renal Kim-1 mRNA is shown (**D**). The panels show mean numbers and fold increase ±standard error of the mean (* *p* < 0.05, control mice versus rTM-treated mice). c/10 hpf, cell numbers per 10 high-power fields. Dotted lines represent mean values from control mice without LPS. Scale bar, 50 μm.

Hematoxylin and eosin staining was performed in a preliminary experiment as a conventional method for evaluating the acute tubular necrosis score. However, there were no apparent optical changes in the damaged proximal tubule epithelial cells. Kidney injury molecule-1 (Kim-1) expression is induced in the apical side of the dilated tubules with damaged proximal tubule epithelial cells during post-repair regeneration [21]. In the previous study, we demonstrated that the numbers of tubular Kim-1+ cells were correlated with the renal damage in AKI [22,23,24,25]. Figure 5 shows tubular Kim-1 expression in LPS-induced AKI. Representative photographs show Kim-1+ cells in the kidney at 48 h (Figure 5A,B). There were more Kim-1+ cells in tubules from the control mice (Figure 5A). In the rTM-treatment mice, Kim-1 was not detected in the kidney (Figure 5B). Figure 5C shows that the number of Kim-1+ cells in rTM-treatment mice was significantly lower than that of the control mice at 48 h. We also investigated Kim-1 mRNA expression and found significantly lower levels of expression in the rTM-treatment mice than in the control mice (Figure 5D).

### 2.7. Renal and Splenic Phospho c-Jun+ Cells

**Figure 6:**
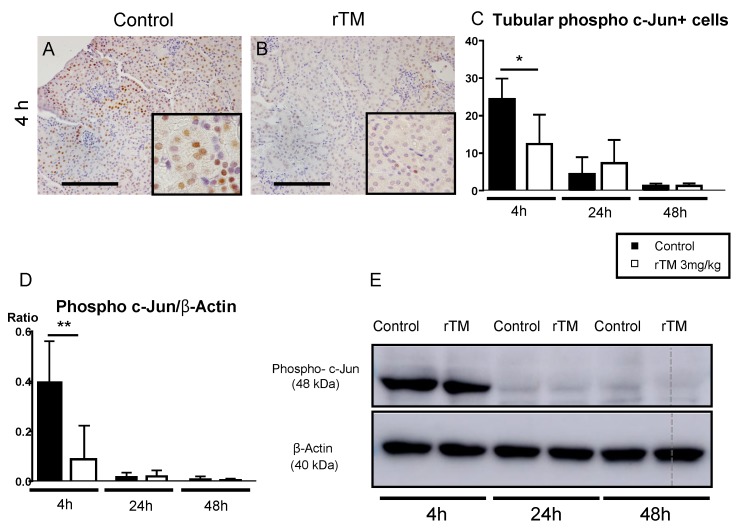
Expression of phospho c-Jun in kidney and spleen cells. Representative photograph of phospho c-Jun expression in the kidney after lipopolysaccharide (LPS) injection (**A**,**B**). In saline-treated control mice, phospho c-Jun-positive tubules were present in the kidney (**A**). Fewer c-Jun-positive cells were seen in rTM-treated mice than in the control mice (**B**). The number of c-Jun-positive tubules in 10 × 400 fields (**C**). Activation of splenic phospho c-Jun were assayed as protein levels by Western blotting (**D**,**E**). The data are the mean ±standard error of the mean (* *p* < 0.05 and ** *p* < 0.01, cell numbers and band density in control versus rTM-treated mice). Scale bar, 5 μm.

In the present study, we also investigated tubular and splenic phospho c-Jun expression in LPS-induced AKI. Representative photographs show phospho c-Jun+ cells in the kidney at 4 h (Figure 6A,B). Interestingly, the number of tubular phospho c-Jun+ cells peaked at 4 h after LPS-injection. After 4 h, the numbers gradually decreased at similar rates in both groups. Figure 6C shows the number of tubular phospho c-Jun+ cells in the rTM-treatment mice, which had decreased significantly compared to that of the control mice at 4 h. We also analyzed splenic phospho c-Jun expression by Western blotting to examine the systemic immune response and found that the expression levels in the rTM-treatment mice were significantly lower compared to that in the control mice at 4 h (Figure 6D,E).

### 2.8. rTM Mediates Apoptosis in Kidney and Tubular Epithelial Cells

**Figure 7:**
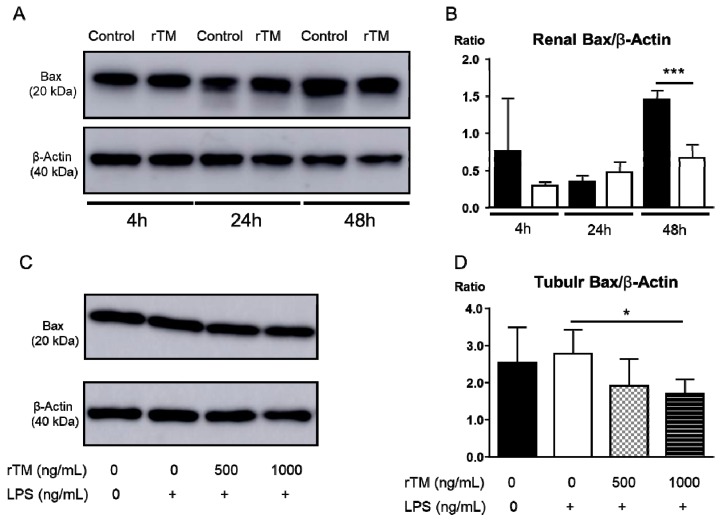
Expression of Bax in the kidney and tubular epithelial cells by Western blotting in vivo and in vitro. Representative photographs show Bax expression in the kidney and tubular epithelial cells (TECs) (**A**,**C**). The ratio of Bax/β-actin in the kidney after lipopolysaccharide (LPS) injection was analyzed at 4, 24, and 48 h (**B**). The ratio of Bax/β-actin in the TECs added with rTM (0, 500, and 1000 ng/mL) in the presence of LPS (300 ng/mL) was analyzed for 24 h (each goup; *n* = 4) (**D**). The data are the mean ± standard error of the mean (*** *p* < 0.001, control versus rTM-treated mice and * *p* < 0.05, LPS 300 ng/mL alone versus rTM 1000 ng/mL + LPS 300 ng/mL).

We evaluated the effect of Bax expression as the apoptosis signal in the kidney and renal tubular epithelial cells (TECs) by exposing LPS by Western blotting in the kidney and TECs in vivo and vitro (Figure 7). In vivo, Bax expression from the protein which was extracted in the whole kidney was significantly lower in the rTM-treatment mice compared to that in the control mice at 48 h after LPS-injection (control 1.5 ± 0.1 versus rTM 0.7 ± 0.1) (Figure 7A,B). In vitro, we also analyzed Bax expression, to see whether rTM could be affected the Bax expression directly in the TECs. Bax expressions in the TECs stimulated with LPS after rTM was added to the group in the concentration of 1000 ng/mL, was significantly lower compared to those with the TEC not added (not added: 2.5 ± 0.4 versus rTM added: 1.7 ± 0.1) (Figure 7C,D).

## 3. Discussion

Soluble TM is increased significantly in the plasma of septic patients [26]. Vincent et al. reported that there were no significant differences in 28-day-all-cause mortality rates and the percentage of renal dysfunction in all of patients in rTM treatment with sepsis-associated coagulopathy [27]. However, the mortality rates in the subgroup of patients with coagulopathy (international normalized ratio > 1.4 and platelet count > 30 × 10^9^/L) were 26.7% in the rTM group and 32.1% in the placebo group, a difference of 5.4%. In the subgroup, there was no statistics analysis in the percentage of renal dysfunction and renal replacement therapy between rTM and placebo groups. However, the potential value of the treatment with rTM in the pathogenesis of LPS-induced renal injury has not been well investigated. We hypothesized that rTM modulated the upregulated expression of cytokines, chemokines, adhesion molecules [28,29,30], and apoptosis pathway seen in the LPS-model, upon which, leukocyte populations, including neutrophils [31,32] and T-cells [33,34], are increased in number and/or are activated. This downstream serum cytokine overexpression ultimately relates to the survival of the animals, and consequently, this exerts a hemodynamic kidney failure or a real structural damage through a kidney inflammatory storm.

The current studies demonstrate that (i) rTM significantly reduces nephrotoxicity after LPS-injection, (ii) rTM affects early immune responses, including activation, and the accumulation of neutrophils and CD4+ T-cells, CD11c+, and macrophages, (iii) rTM ameliorates the LPS-induced upregulation of proinflammatory cytokines and chemokines, resulting in decreased infiltration of leukocytes into the kidney, and (iv) the protection from nephrotoxicity is mediated through phospho c-Jun expression in the systemic immune system in the splenocytes.

Thrombomodulin is a high-affinity thrombin receptor on the endothelial cell membranes and plays an important role as a natural anticoagulant. It acts as a cofactor for the thrombin-catalyzed activation of protein C and inhibits the procoagulant functions of thrombin. It is located in other various cell types (e.g., keratinocytes, osteoblasts, macrophages, etc.), in which it is thought to be involved in cell differentiation or inflammation. In the presence of cytokines, activated neutrophils, and macrophages, endothelial TM is enzymatically cleaved, circulating in the blood and releasing soluble fragments that are excreted in the urine.

In the context of AKI, epithelial and endothelial cells are activated and upregulate the production of various cytokines (such as TNF-α) that help initiate the inflammatory response [35,36]. Exposure of the endothelium to TNF-α and IL-1β leads to the downregulation of TM expression, which in turn is downregulated by numerous factors, including TNF-α and IL-1β, endotoxin, tissue growth factor-β, hypoxia, oxidized low-density lipoprotein, shear stress, and oxidation of Met388 residue on the glycoprotein [37]. The resulting endothelial dysfunction exacerbates microvascular congestion, stasis, and the interaction of continuous activated vascular endothelial cells with leukocyte adhesion and subsequent microcirculatory abnormalities [38,39]. Furthermore, Conway et al. reported that the *N*-terminal lectin-like domain of TM, which lacks anticoagulant function, causes a downregulation of expression of inflammatory cell adhesion molecules, such as intercellular adhesion molecule and selectins, leading to decreased inflammatory cell adhesion, infiltration, and cytokine release [40]. A previous study has also shown that TM knockout in macrophages reduces LPS binding to macrophages and suppresses LPS-induced inflammation [41]. In accordance with our previously published report [42], the importance of TNF, IFN-γ, and IL-18 as pro-inflammatory cytokines has been demonstrated in the pathogenesis of AKI after LPS-injection, and we observed a significant reduction in the expression of these cytokines in LPS-induced AKI. In the current study, we demonstrated that rTM attenuated renal damage by reducing cytokine and apoptotic signals with the accumulation of CD4+ T-cells, CD11c+ cells, and F4/80+ cells in LPS-induced AKI. Indeed, AKI enhanced phospho c-Jun expression, the levels of which greatly increased following LPS-injection. Apoptosis plays a causative role in the pathogenesis of AKI [43,44], and c-Jun *N*-terminal kinase (JNK), a stress-activated protein kinase, plays a critical role in the type of apoptosis mediated by mitochondrial dysfunction [45]. The transcriptional activity of c-Jun is regulated by phosphorylation at Ser63 and Ser73 via JNK [46]. Recent studies have elucidated the roles played by JNK in AKI [47,48]. Notably, JNK was strongly associated with heat shock protein 70 (Hsp70), thereby inhibiting its phosphorylation in rTM-treated LPS-induced AKI. Among several Hsp70 kinases, JNK stimulated by cellular stresses phosphorylates Hsp70 at the serine residue and promotes its dissociation from numerous binding partners, including Bax [49]. This in turn leads to the mitochondrial translocation of these binding partners and thereby promotes apoptotic cell death. Thus, the manipulation of the JNK signaling pathway may affect the severity of AKI.

Bax is known as an apoptotic promotion marker. The binding of Fas and FasL leads to the activation of caspases and subsequent apoptosis. Bax translocation is dependent on caspase activation, and it increases the release of cytochrome c from mitochondria, thereby promoting apoptosis [50]. Kim-1 expression is also induced in damaged proximal tubule epithelial cells during post-repair regeneration [21]. In the event of kidney damage, intramembrane cleavage at the cell surface causes Kim-1 to be shed into the lumen of the renal tubule. Its renal expression has been associated with the dedifferentiation of epithelial cells [21] and Kim-1 allows tubular cells to participate in the phagocytosis of apoptotic debris via recognizing phosphatidylserine [51]. In the current study, the numbers of tubular Kim-1+ cells at 24 h were no different between the two groups. At 48 h, the numbers of Kim-1+ cells were increased in control mice compared that in rTM mice. Furthermore, kidney functions in the two groups were recovered at 48 h. We consider that Kim-1 acts as a receptor for the eat-me signal that appears on the surface of apoptotic cells and is thought to help with elimination of apoptotic proximal tubule epithelial cells into the lumen during the repair process. These findings suggested that tubular Kim-1 expressions were correlated with Bax expressions as an apoptosis marker in the kidney.

We demonstrated the significantly lower levels in the expression of inflammatory cytokines and renal cytokine mRNA expression (especially that of IL-18, IFN-γ, TNF, and IL-6) in rTM-treatment mice compared to the control mice. Moreover, we demonstrated the suppression of the expected accumulation of CD4+ T-cells, F4/80+, and CD11c+ cells such as APCs in rTM-treatment mice. The treatment with rTM also suppressed phospho c-Jun expression through the JNK pathway.

In conclusion, rTM treatment attenuated kidney injury and improved survival rates following LPS-induced AKI in mice. The mechanism underlying these effects of TM may be mediated by a reduction in inflammatory cytokine production via the JNK pathway in response to LPS exposure.

## 4. Materials and Methods

### 4.1. Ethics Statement

The animal protocols were approved by the Kindai University Animal Care Committee (29-003; 03/01/2017) and were performed in accordance with the Kindai University Animal Care Guidelines.

### 4.2. Animals

The C57BL/6 mice used for this experiment were purchased from Shizuoka Laboratory Animal Centre (Shizuoka, Japan). All mice were maintained in our specific pathogen-free animal facility.

### 4.3. Murine Model of Endotoxin-Induced Acute Kidney Injury

In all experiments, 8–10-week-old male mice were injected i.p. with 15 mg/kg LPS (*Escherichia coli* O111:B4, Sigma-Aldrich, St. Louis, MO, USA) at 0 h. This dose was chosen on the basis of our preliminary studies showing that higher doses did not induce consistent and significant renal dysfunction and tubular injury. Before the 3 and 24 h LPS-injection, the mice were injected i.p. twice with saline as a control group, while rTM (Asahi Kasei Pharm Co., Tokyo, Japan; 3 mg/kg, i.p.) was administered to the animals in the treatment group. Blood was collected in heparinized tubules for the measurement of BUN, TNF, IFN-γ, IL-10, and IL-18. Mice were sacrificed at 0 (rTM and control, *n* = 3), 4 (*n* = 6 and 7), 24 (*n* = 5 and 4) and 48 h (*n* = 6 and 5) after induction of AKI with the collection of blood, as described above, and the harvesting of kidney and spleen tissue. The resulting lethality was monitored for 48 h after LPS-injection. The BUN at 0, 24, and 48 h was measured by an autoanalyzer (Hitachi, Tokyo, Japan).

### 4.4. Assessment of Renal Injury

Immunohistochemical staining for CD4+ T-cells, Gr-1+ cells as neutrophils, and tubular Kim-1 (kidney injury molecule-1) was performed on 6 μm periodate lysine paraformaldehyde-fixed sections [36]. Here, CD11c+ cells and F4/80+ cells were examined as antigen-presenting cells (APCs; DCs, CD11c+ and pan-macrophages, F4/80+ cells), and phospho c-Jun+ cells were identified in 4 μm-thick formalin-fixed sections. The numbers of these cells were assessed in 10 fields per slide at ×400 magnification, and the results are expressed as cells per high-power field (c/hpf). The primary monoclonal antibodies used were rat monoclonal antibody GK1.5 for CD4+ T-cells (Pharmingen, San Diego, CA, USA), F4/80 hybridoma culture supernatant (HB 198; American Type Culture Collection, Manassas, MD, USA), mouse monoclonal antibody for CD11c+ cells (Abcam, Cambridge, U.K.), RB6–8C5 for neutrophils (anti-Gr-1; DNAX, Palo Alto, CA), and rat monoclonal antibody Tim-1 (R&D Systems, Minneapolis, MN, USA). Isotype-matched irrelevant monoclonal antibodies were used as controls.

### 4.5. Measurement of mRNA Expression in the Kidney by Real-Time PCR

For the measurement of the mRNA expression of TNF, the CCL2/MCP-1, GATA3, T-bet, and 18SrRNA by FastStart DNA Master SYBR Green I (Applied Biosystems, Foster City, CA, USA) and IFN-γ, IL-10, IL-18, Kim-1, TLR4 (Toll-like receptor4), and 18SrRNA by TaqMan gene (Applied Biosystems) on whole kidney tissue, we performed a real-time PCR as described [36]. The sequences of the primer and gene database numbers are listed in Table 2 and Table 3. The relative amount of mRNA was calculated using the comparative Ct (∆∆Ct) method. All specific amplicons were normalized against 18SrRNA, which was amplified in the same reaction as an internal control using commercial reagents (Applied Biosystems) and values are expressed as the fold-increase relative to those of saline-treated control mice.

### 4.6. Serum Cytokine Quantitation by Enzyme-Linked Immunosorbent Assay

Serum TNF, IFN-γ, IL-10, and IL-18 levels were determined by an enzyme-linked immunosorbent assay kit for each cytokine (BD Biosciences, San Diego, CA, USA), as previously described [44,45].

### 4.7. Cell Culture

TECs were removed from C57BL/6 mice at the age of 8–10 weeks with no treatment of LPS and rTM, and single-cell suspensions were made in RPMI 1640 medium supplemented with 10% fetal calf serum and adjusted to 2 × 10^6^ cells/well. TECs were isolated for primary culture with minor modifications, as described previously [46,47]. The isolated TECs were grown for 6–7 days and then plated at 100 mm dish for experiments. Then, TECs were incubated with rTM (0, 500, and 1000 ng/mL) in the presence of LPS (300 ng/mL) or medium alone in fresh culture medium for 24 h. After incubation, TECs were analyzed in Western blotting analysis.

### 4.8. Western Blotting Analysis

Proteins were extracted by homogenization of the kidney at 4, 24, and 48 h after LPS-injection and TECs in tissue protein extraction reagent (Pierce, Rockford, IL, USA) to determine Bax, as described elsewhere [47]. Monoclonal anti-β-actin antibody was obtained from Santa Cruz Biotechnology (St. Louis, MO, USA). Anti-mouse Bax antibody was obtained from Cell Signaling Technology (Danvers, MA, USA). Peroxidase-conjugated goat immunoglobulin G was purchased from Santa Cruz Biotechnology.

### 4.9. Statistical Analysis

The results are expressed as the mean ± standard error of the mean Groups were compared by the unpaired t-test or by an analysis of variance (ANOVA), when more than two groups were compared. Probability values < 0.05 were accepted as significant. The survival time was estimated using the Kaplan–Meier method. The log-rank test was used to compare survival times between groups.

## Figures and Tables

**Table 1:** Effect of recombinant human soluble thrombomodulin (rTM) on gene expression in lipopolysaccharide (LPS)-induced acute kidney injury.

Gene Expressions	4 h	24 h	48 h
	Control versus rTM	Control versus rTM	Control versus rTM
Cytokines			
IL-10	36.5 ± 3.9 versus 35.8 ± 7.3	84.0 ± 19.1 versus 91.4 ± 17.8	31.1 ± 9.6 versus 54.6 ± 9.4 *
IL-18	2.4 ± 0.2 versus 2.9 ± 0.5	2.9 ± 0.4 versus 4.4 ± 0.5	1.3 ± 0.5 versus 1.2 ± 0.3
IFN-γ	8.6 ± 1.5 versus 11.4 ± 4.4	2.1 ± 0.5 versus 1.5 ± 0.2 *	0.5 ± 0.1 versus 0.8 ± 0.2
TNF	12.7 ± 1.6 versus 8.7 ± 1.8 *	10.8 ± 4.7 versus 4.3 ± 0.6 *	2.8 ± 0.3 versus 3.3 ± 0.3
Chemokines			
CCL2/MCP-1	245.9 ± 27.9 versus 187.2 ± 30.1 *	38.1 ± 4.9 versus 25.8 ± 2.4 *	17.0 ± 3.1 versus 24.4 ± 3.6
TLR4	1.9 ± 0.3 versus 2.1 ± 0.3	1.1 ± 0.2 versus 1.9 ± 0.6	1.3 ± 0.1 versus 1.7 ± 0.3
Th-cell subset transcription factors			
GATA3	0.6 ± 0.2 versus 1.0 ± 0.4	0.3 ± 0.1 versus 0.3 ± 0.1	0.5 ± 0.1 versus 0.6 ± 0.5
T-bet	6.5 ± 3.4 versus 0.8 ± 0.6 *	0.3 ± 0.1 versus 0.3 ± 0.1	0.3 ± 0.2 versus 0.2 ± 0.1

The gene expressions of cytokines, chemokines and Th cell subset transcription factors were measured in control and rTM-treatment mice at 4, 24, and 48 h after LPS injection by real-time reverse transcription-polymerase chain reaction In each experiment, the expression levels were normalized to the expression of 18SrRNA and are expressed relative to the values of saline-treated control mice. The data are the mean fold-increase ±standard error of the mean (* *p* < 0.05). IL, interleukin; IFN, interferon; TNF, tumor necrosis factor; CCL2/MCP-1, chemokine (C-C motif) ligand 2/monocyte chemoattractant protein-1; TLR, toll like receptor.

**Table 2:** Primer sequences for analysis of mRNA expression.

Gene Expression	Forward Primer	Reverse Primer
18SrRNA	GTAACCCGTTGAACCCCATTC	GCCTCACTAAACCATCCAATCG
TNF	CGATCACCCCGAAGTTCAGTA	GGTGCCTATGTCTCAGCCTCTT
CCL2/MCP-1	AAAAACCTGGATCGGAACCAA	CGGGTCAACTTCACATTCAAAG
GATA3	AGGGACATCCTGCGCGAACTGT	CATCTTCCGGTTTCGGGTCTGG
T-bet	CCTGGACCCAACTGTCAACT	AACTGTGTTCCCGAGGTGTC

TNF, tumor necrosis factor; CCL2, chemokine (C-C motif) ligand 2; MCP-1, monocyte chemoattractant chemokine-1.

**Table 3:** Gene database number for analysis of mRNA expression.

Gene Expression	Gene Database Number
18SrRNA	NM_026744.3
IFN-γ	NM_008337.3
IL-10	NM_010548.1
IL-18	NM_008360.1
Kim-1	NM_134248.1
TLR4	Mm00445273_m1

IFN, interferon; IL, interleukin; Kim, kidney injury molecule; TLR4, toll-like receptor 4.

## Abbreviations

AKI	acute kidney injury
IL	interleukin
TNF	tumor necrosis factor
TM	thrombomodulin
rTM	recombinant human soluble TM
APC	anticoagulant protein C
LPS	lipopolysaccharide
IFN-γ	interferon-gamma
i.p.	intraperitoneally
BUN	blood urea nitrogen
CCL2/MCP-1	chemokine ligand 2/monocyte chemoattractant protein-1
Kim-1	kidney injury molecule-1
TEC	tubular epithelial cells
APCs	antigen-presenting cells
TLR4	toll-like receptor4
JNK	c-Jun *N*-terminal kinase
Hsp70	heat shock protein 70
ANOVA	analysis of variance
